# Assessment of psychometric properties of the Brazilian version of the oral anticoagulation knowledge test

**DOI:** 10.1186/s12955-016-0498-3

**Published:** 2016-06-24

**Authors:** Marcus Fernando da Silva Praxedes, Mauro Henrique Nogueira Guimarães de Abreu, Saul Martins Paiva, Juliana Vaz de Melo Mambrini, Milena Soriano Marcolino, Maria Auxiliadora Parreiras Martins

**Affiliations:** Programa de Pós-Graduação em Medicamentos e Assistência Farmacêutica. Faculdade de Farmácia, Universidade Federal de Minas Gerais (UFMG), Belo Horizonte, Brazil; Departamento de Odontologia Social e Preventiva, Faculdade de Odontologia, Universidade Federal de Minas Gerais, Av. Antônio Carlos, 6627 Pampulha, CEP 31270-901 Belo Horizonte, MG Brazil; Departamento de Odontopediatria e Ortodontia, Faculdade de Odontologia, Universidade Federal de Minas Gerais, Belo Horizonte, Brazil; Centro de Pesquisas René Rachou, Fundação Oswaldo Cruz, Belo Horizonte, Brazil; Departamento de Clínica Médica, Faculdade de Medicina, Universidade Federal de Minas Gerais, Belo Horizonte, Brazil

**Keywords:** Anticoagulants, Validation studies, Patient knowledge about medication, Questionnaires

## Abstract

**Background:**

The aim of this study was to evaluate the psychometric properties of the Brazilian version of the *Oral Anticoagulation Knowledge (OAK) Test*.

**Methods:**

This study, conducted in an anticoagulation clinic, included 201 Brazilian participants aged over 18 years, who had been using warfarin for more than two months. The reliability of the instrument was evaluated by assessing internal consistency (Kuder-Richardson coefficient) and reproducibility (test-retest reliability). The validity was evaluated by hypothesizing that there would be a positive correlation of moderate to strong intensity between the correctness levels of the OAK Test and *time within therapeutic range* (TTR) values, which is a measure used to evaluate the quality of oral anticoagulation.

**Results:**

The instrument exhibited good psychometric properties. The total a Kuder-Richardson coefficient value was 0.818 and intraclass correlation coefficient was 0.967. The validity revealed a strong positive correlation between the values of the level of knowledge, as measured by the OAK Test and the TTR values (r_s_ = 0.780).

**Conclusion:**

The instrument proved to be a reliable and valid tool for evaluating the knowledge of Brazilian patients on oral anticoagulation therapy with warfarin. This instrument may be incorporated into the practice of health care for substantiating the structuring of educational activities to ensure the improvement of knowledge about the use of warfarin, thereby increasing the effectiveness and safety of treatment.

## Background

Warfarin is an oral anticoagulant, coumarinic derivative used worldwide for primary and secondary prophylaxis of thromboembolism. Its efficacy and safety have been widely studied for several indications through well-conducted clinical trials. The indication of oral anticoagulation is increasing due to population aging and the consequent increase in the incidence of thromboembolic diseases [1–5].

The management of warfarin is quite complex due to its narrow therapeutic range and wide dose–response variability. Among the interfering factors in the anticoagulant effect, we can cite the presence of genetic polymorphisms, drug and dietary interactions of potential clinical significance, as well as changes in the patient’s condition as some of the common ones [1, 6].

For proper dose management, laboratory monitoring is conducted by means of the International Normalized Ratio (INR) calculated from the prothrombin activity. The target INR is considered between 2.00 and 3.00 for most of warfarin indications. Subtherapeutic results were related to the occurrence of venous thromboembolism and higher values, to increased risk of bleeding [1]. To evaluate the quality of anticoagulant therapy, the percentage of time of INR in the therapeutic range is calculated (*time within therapeutic range;* TTR), the bleeding and mortality rates being significantly higher in patients with TTR values <60 %, in comparison with those presenting TTR values >75 % [7].

Studies have indicated that higher levels of knowledge regarding warfarin therapy are related to an improved stability of INR and increased TTR values [6, 8]. It is therefore essential that patients using warfarin have adequate knowledge about the therapeutic objective (indication and effectiveness), the process of use (dosage, therapeutic regimen, route of administration, and duration of treatment), safety (adverse events, precautions, contraindications, and interactions), as well as the conservation of the drug [9]. In this sense, the evaluation of the patient’s level of knowledge using validated instruments becomes necessary for identifying deficiencies and guide educational activities to create a continuous system of quality improvement of patient monitoring and safety [10].

Among the few validated tools to evaluate patients’ knowledge regarding the use of warfarin, we can highlight the *Oral Anticoagulation Knowledge* (OAK) Test, which was validated for use in the United States of America [8] and Malaysia [11], and showed good psychometric properties. The OAK Test was cross-culturally adapted into Brazilian Portuguese, presenting a semantic and conceptual equivalence of items in relation to the original version [12]. It is recommended that the psychometric properties of this adapted version be evaluated to ensure its validity and reliability to achieve the proposed objectives [13]. Therefore, the present study aimed at evaluating the psychometric properties of the Brazilian version of the OAK Test.

## Methods

### Study design and setting

This is a methodological study designed according to the recommendations outlined in the literature for the evaluation and validation of the instrument in question [14]. This study was conducted in an anticoagulation clinic of a university hospital located in Belo Horizonte, southeastern Brazil, which is a regional reference center that offers medium and high complexity health assistance.

### Sample

The number of participants in the phases of test and retest of the questionnaire was according to the recommendation to include at least 10 respondents for each question of the instrument under validation and 30 % of the initial sample for retest [15]. Thus, the sample comprised 201 consecutive patients who were recruited during the service office hours, were aged 18 years or older, were Brazilians, and were on warfarin for at least one year. For the retest, 60 of these patients were randomly selected for a new interview that was conducted within two to three months after the first interview, a period equal to that used in the study in which the original version of the OAK *Test* was developed [8]. It is noteworthy that the correct answers to the test were not revealed to the participants during any of the steps of the study.

### The instrument

The psychometric properties of the Brazilian version of the OAK Test [12] were evaluated in this study by employing internationally recognized methodology [13, 16]. The instrument consists of 20 questions with four possible answers, and only one correct option. Each correct answer equals one point, leading to a total score ranging from zero to 20 points. A higher score indicates better level of knowledge about oral anticoagulant therapy. Content and face validity were evaluated by an expert committee that examined the relevance of each item of the instrument and their effectiveness in measuring what they propose to measure, respectively [12].

### Evaluation of psychometric properties

The evaluation of the psychometric properties of the Brazilian version of the OAK Test included the analysis of reliability and validity. The instrument’s reliability was evaluated by assessing the internal consistency (Kuder-Richardson coefficient) and reproducibility (test-retest reliability).

For the analysis of the validity, it was hypothesized that there would be a positive correlation, of moderate to strong intensity, between the correctness levels of the OAK Test and the TTR values.

### Data collection

Data were collected between January to April 2015 by means of individual interviews and consultation of the hospital records of the participants. Instruments were applied for sociodemographic (age, sex, education, and income) and clinical characterization (TTR), and the Brazilian version of the OAK Test was administered. Interviews were conducted in a standardized manner by a single interviewer to avoid interference with the respondents’ answers. For TTR calculation, all patients’ INR values obtained up to one year prior to interview were interpolated by using the method proposed by Rosendaal. This method assumes that there is a linear variation between values over time and the result correspond to the percentage of time that anticoagulation has been within therapeutic range [17].

### Statistical analysis

The database was validated by double entry in the *EpiData* program (version 3.1, EpiData Assoc, Denmark) and analyzed using the *Statistical Package for Social Sciences* (SPSS for Windows, version 20.0, SPSS Inc., Chicago, IL). Descriptive analyses were performed for all variables using measures of dispersion, frequency, and central tendency.

Statistical analysis involved the evaluation of internal consistency by the Kuder-Richardson coefficient, for which values above 0.70 reflect a high degree of internal consistency [15, 18]. Temporal stability was verified by the values of the intraclass correlation coefficients (ICC) of the OAK final score (quantitative variable with scores varying from zero to 20) and by calculating the *Cohen’s Kappa* coefficients for each dichotomized question of the OAK Test (correctness and error).

We analyzed the *Spearman* correlation between the OAK Test correctness levels and TTR to evaluate validity. The results were analyzed according to the following classification: values below 0.30, even when statistically significant, are of little clinical applicability, those between 0.30 and 0.50 are moderate, and those above 0.5 are of strong magnitude [19].

We also evaluated the correlations between the OAK Test and age (years), educational level (years) and income (Brazilian *Reais*). The gender-based comparison of the OAK Test values was conducted using the *Mann–Whitney* test, considering that the instrument did not show normal distribution (*Kolmogorov-Smirnov* test, *p <* 0.001). The statistical significance was declared at *p =* 0.05.

The description of knowledge was categorized according to previous studies that used the same instrument validated in other countries. The categories have included scores <50 % as low, 50–75 % as average, and >75 % as high level of knowledge regarding oral anticoagulation [11, 20]. The quality of oral anticoagulation was evaluated by stratified TTR, according to a previous study, classified into three groups: <60 %, 60 %–75 % and >75 % [7].

## Results

The final analysis included 201 participants under treatment with warfarin. The characteristics of the patients have been presented in Table 1. The average number of correct answers to the OAK Test was 63 % (±22) (Table 1). The analysis of the instrument’s internal consistency revealed a Kuder-Richardson coefficient of 0.818 for the total score. The exclusion of any instrument items showed small changes in the Kuder-Richardson coefficient, which ranged from 0.802 to 0.821 (Table 2).

**Table 1:** Sociodemographic and clinical characteristics, Brazil, 2015

Characteristics	Total sample *N =* 201
Age, mean (SD)	55 (13)
Age group, n	
<45	36 (18)
45–60	87 (43)
61–75	64 (32)
≥76	14 (7)
Sex, n	
Female	122 (60)
Education, n	
No formal education	8 (4)
Incomplete primary education	126 (63)
Complete primary education	28 (14)
High school	37 (18)
University	02 (1)
TTR (% INR control), n	
TTR < 60 %	94 (47)
TTR 60–75 %	51 (25)
TTR > 75 %	56 (28)
Monthly income (USD), mean (SD)	416 (238)
OAK’s questions answered correctly, mean (SD)	63 (22)
OAK categories	
<50 % (low level of knowledge)	59 (29)
50–75 % (moderate level of knowledge)	83 (42)
>75 % (high level of knowledge)	76 (29)

*OAK,* oral anticoagulation knowledge; SD, standard deviation; TTR, *time within therapeutic range*

**Table 2:** Percentage of correct answers in Brazilian version of OAK *Test* and the correlation values after exclusion of each item (*n =* 201), 2015

Brazilian version of *OAK Test* (Kuder-Richardson coefficients total = 0.818)	Percentage of correct answers	Kuder-Richardson coefficients if item deleted
1. Missing one dose of Coumadin (warfarin): 1. *Esquecer de tomar uma dose da varfarina:* Correct answer: a. has no effect **b. can alter the drug’s effectiveness** ^a^ c. is permissible as long as you take a double dose the next time d. is permissible as long as you watch which foods you eat	84 %	0.821
2. You can distinguish between different strengths of Coumadin (warfarin) tablets by what? *2. Você consegue diferenciar entre diferentes doses do comprimido da varfarina utilizando-se de?* Correct answer: **a. color** b. shape c. size d. weight	78 %	0.818
3. A patient on Coumadin (warfarin) therapy should contact the physician or healthcare provider who monitors it when: 3. *O paciente que toma varfarina deve entrar em contato com o médico ou quem acompanha o tratamento quando:* Correct answer: a. another physician adds a new medication b. another physician stops a current medication c. another physician changes a dose of a current medication **d. all of the above**	63 %	0.803
4. Occasionally eating a large amount of leafy greens vegetables while taking Coumadin (warfarin) can: 4. *Ocasionalmente comer uma grande quantidade de folhas verdes enquanto toma varfarina pode*: Correct answer: a. increase your risk of bleeding from Coumadin (warfarin) **b. reduce the effectiveness of the Coumadin (warfarin)** c. cause upset stomach and vomiting d. reduce your risk of having a blood clot	51 %	0.815
5. Which of the following vitamins interacts with Coumadin (warfarin)? 5. *Qual das vitaminas abaixo interage com a varfarina?* Correct answer: a. vitamin B12 b. vitamin A c. vitamin B6 **d. vitamin K**	49 %	0.808
6. When is it safe to take a medication that interacts with Coumadin (warfarin)? 6. *Quando é seguro tomar um medicamento que interage com a varfarina?* Correct answer: a. if you take the Coumadin (warfarin) in the morning and the interacting medication at night **b. if your healthcare provide is aware of the interaction and checks your PT/INR (“Protime”) regularly** c. if you take your Coumadin (warfarin) every other day d. it is never safe to take a medication that interacts with Coumadin (warfarin)	40 %	0.804
7. The PT/INR (“Protime”) test is: 7. *O exame de RNI é:* Correct answer: **a. a blood test used to monitor your Coumadin (warfarin) therapy** b. a blood test that is rarely done while on Coumadin (warfarin) c. a blood test that checks the amount of vitamin K in your diet d. a blood test that can determine if you need to be on Coumadin (warfarin)	88 %	0.818
8. Coumadin (warfarin) may be used to: 8. *A varfarina pode ser usada para:* Correct answer: **a. treat people that already have a blood clot** b. treat people that have high blood sugar levels c. treat people with high blood pressure d. treat people with severe wounds	91 %	0.813
9. A patient with a PT/INR (“Protime”) value below their “goal range”: 9. *Um paciente com o RNI abaixo da “faixa desejada”:* Correct answer: a. is at an increase the risk of bleeding **b. is at an increase the risk of having a clot** c. is more likely to have a skin rash from the Coumadin (warfarin) d. is more likely to experience side effects from Coumadin (warfarin)	67 %	0.803
10. Taking a medication containing aspirin or other non-steroidal anti-inflammatory medications such as ibuprofen (Motrin®/Advil®) while on Coumadin (warfarin) will: 10. *Tomar um medicamento que contenha ácido acetilsalicílico (AAS) ou outros anti-inflamatórios não esteroides, como ibuprofeno, enquanto estiver tomando a varfarina irá:* Correct answer: a. reduce the effectiveness of the Coumadin (warfarin) **b. increase your risk of bleeding from the Coumadin (warfarin)** c. cause a blood clot to form d. require you to increase your dose of Coumadin (warfarin)	46 %	0.805
11. A person on Coumadin (warfarin) should seek immediate medical attention: 11. *Uma pessoa que toma varfarina deve procurar atendimento médico imediatamente:* Correct answer: a. if they skip more than two doses of Coumadin (warfarin) in a row **b. if they notice blood in their stool when going to the bathroom** c. if they experience a minor nosebleed d. if they develop bruises on their arms or legs	50 %	0.807
12. Skipping even one dose of your Coumadin (warfarin) can: 12. *Deixar de tomar uma única dose da varfarina pode:* Correct answer: a. cause your PT/INR (“Protime”) to be above the “goal range” b. increase your risk of bleeding **c. cause your PT/INR(“Protime”) to be below the “goal range”** d. decrease your risk of having a clot	58 %	0.811
13. Drinking alcohol while taking Coumadin (warfarin): 13. *Ingerir bebidas alcoólicas enquanto estiver em tratamento com a varfarina*: Correct answer: a. is safe as long as you separate your dose of Coumadin (warfarin) and the alcohol consumption **b. may affect your PT/INR (“Protime”)** c. does not affect your PT/INR (“Protime”) d. is safe as long as you are on a low dose	84 %	0.819
14. Once you have been stabilized on the correct dose of Coumadin (warfarin), about how often should your PT/INR (“Protime”) value be tested? 14. *Uma vez que você tenha estabilizado sua dose correta da varfarina, com que frequência o valor do seu RNI deve ser testado?* Correct answer: a. once a week **b. once a month** c. once every other month d. once every 3 months	65 %	0.816
15. It is important for a patient on Coumadin (warfarin) to monitor for signs of bleeding: 15. *É importante para um paciente em uso da varfarina estar atento a sinais de sangramento:* Correct answer: a. only when their PT/INR (“Protime”) is above the goal range **b. at all times** c. only when their PT/INR (“Protime”) is below the goal range d. only when you miss a dose	56 %	0.804
16. The best thing to do if you miss a dose of Coumadin (warfarin) is to? 16. *A melhor coisa a ser feita se você esquecer de tomar uma dose da varfarina é?* Correct answer: a. double up the next day **b. take the next scheduled dose and tell your healthcare provider** c. call your healthcare provider immediately d. discontinue Coumadin (warfarin) altogether	74 %	0.810
17. When it comes to diet, people taking Coumadin (warfarin) should: 17. *Quando se trata da alimentação, as pessoas que tomam varfarina devem:* Correct answer: a. never eat foods that contain large amounts of vitamin K b. keep a diary of all of the foods they eat **c. be consistent and eat a diet that includes all types of food** d. increase the amount of vegetables they eat	39 %	0.806
18. Each time you get your PT/INR (“Protime”) checked, you should: 18. *Cada vez que você fizer seu exame RNI, você deve:* Correct answer: a. skip your dose of Coumadin (warfarin) on the day of the test b. avoid eating high fat meals on the day of the test c. avoid foods high in vitamin K on the day of the test **d. let your doctor know if you missed any doses of Coumadin (warfarin)**	44 %	0.807
19. Which of the following over-the-counter products is most likely to interact with Coumadin (Warfarin)? 19. *Qual dos seguintes produtos, que não precisam de receita, é mais provável de interagir com a varfarina?* Correct answer: a. nicotine replacement therapies **b. herbal/dietary supplements** c. allergy medications d. calcium supplements	58 %	0.813
20. A patient with a PT/INR (“Protime”) value above the “goal range”: 20. *Um paciente com um valor de RNI acima da “faixa desejada”:* Correct answer: a. is at an increased risk of having a clot b. is more likely to have drowsiness and fatigue from Coumadin (warfarin) **c. is at an increased risk of bleeding** d. is less likely to experience side effects from Coumadin (warfarin)	73 %	0.802

^a^The correct answer for each question is highlighted in bold. *OAK* Oral Anticoagulation Knowledge, *INR* international normalized ratio, *PT prothrombin time*

The intraclass correlation coefficient of the total score of the OAK Test was 0.967. Table 3 shows the *Cohen's Kappa* coefficients of the test and retest performed, revealing temporal stability ranging between good and perfect. By using a specific evaluation form, most items of the instrument, 19 (95 %), were considered relevant for the study population. Only item 2 was regarded irrelevant to the study in question, but it was maintained in the instrument.

**Table 3:** Temporal stability of questions Brazilian version of OAK Test, 2015

Question (right/error)	Cohen’s Kappa coefficients
Question 1	0.839
Question 2	0.762
Question 3	1
Question 4	0.764
Question 5	0.834
Question 6	0.933
Question 7	0.743
Question 8	1
Question 9	0.761
Question 10	0.867
Question 11	1
Question 12	0.857
Question 13	0.743
Question 14	0.960
Question 15	0.965
Question 16	0.847
Question 17	0.794
Question 18	0.834
Question 19	0.694
Question 20	0.841

The validity revealed a strong positive correlation between the values of the level of knowledge measured by the OAK Test and the TTR (r_s_ = 0.780; *p =* 0.001). There was a positive and weak correlation between the OAK Test score and education (r_s_ = 0.191; *p =* 0.007) and income (r_s_ = 0.170; *p =* 0.016). Other analyses were performed and showed a negative but weak and non-significant correlation between the OAK Test score and age (r_s_ = −0.116; *p =* 0.100). There was no association between the OAK Test score and the gender of the participants (*Mann–Whitney* Test, *p =* 0.182).

## Discussion

This study systematically evaluated the psychometric properties of the Brazilian version of the OAK Test [12]. The results of this evaluation demonstrated adequate performance of the instrument, supporting its validation for use in Brazil.

The reliability of an instrument is related to the ability to measure an attribute accurately, over time, and the consistency and stability of the attribute measured [19]. In this sense, the Brazilian version of the OAK Test retained the reliability and validity presented by the original English instrument. Reliability was confirmed by internal consistency and test-retest measures and the content, face [12], and validities were also confirmed.

In terms of the application of the OAK Test, good internal consistency was observed as the temporal stability ranged between good and perfect. These results demonstrate that the instrument in question is reliable and has adequate temporal stability, corroborating the findings of the original study in which the instrument was developed [8], and another study that obtained similar results [11].

Through the initial hypothesis that there would be a positive correlation, of moderate to strong intensity, between the correctness levels of the OAK Test and the TTR values, the validity of the instrument was proven, since there was a strong positive correlation (r_s_ = 0.780). This hypothesis was also confirmed by other investigations, demonstrating that the greater the patient’s knowledge about treatment with warfarin, the better was the control of oral anticoagulation, as expressed by higher TTR values [11, 20]. We observed that formal education and income showed a weak association with the OAK Test, which is similar to the results of other studies that have not demonstrated significant associations between knowledge and these variables as well [8, 21].

By applying the OAK Test, it was possible to verify the level of knowledge of the patients treated with warfarin in a public hospital in Brazil. The average correctness of the test answers was 63 %, which is in line with that found in other studies [8, 22]; besides 71 % of patients had average or high level of knowledge. However, a similar study conducted in Malaysia identified an average of only 11.2 % of correctness [11]. Studies that used other tests also revealed differences in the level of knowledge of participants. Both low levels of knowledge [23, 24], and good levels of knowledge were observed [10, 25]. Such differences may be related to sociodemographic and clinical particularities of the patients included, the quality of anticoagulation services, or methodological differences employed in the application of knowledge tests.

In the present study, it was observed that a deficit in knowledge about the drug and dietary interactions with warfarin correlated with a correctness rate of less than 50 %. These data are in line with those of another study carried out in an anticoagulation clinic, which showed meaningful gaps in the knowledge regarding the drug and dietary interactions [22]. These findings signal the importance of implementing educational strategies to improve the understanding of these issues using approaches that can contribute to modifying patients’ behavior regarding self-care and appropriate lifestyle choices.

Most patients had TTR lower than 60 %, which is a worrisome result since the rates of bleeding and mortality are significantly higher in patients with TTR < 60 % when compared to those with TTR > 75 % [7]. Laboratorial monitoring is necessary to guide dose adjustments in order to achieve INR target. Interventions focused on INR control, and consequent TTR elevation, contribute to the increase of effectiveness of drug therapy and the minimization of adverse drug events. In this sense, the use of valid and reliable instruments allow health teams to evaluate the level of knowledge of patients and identify knowledge deficits on important aspects related to the treatment with warfarin substantiating appropriate decision for patient management [1].

Regarding the limitations of this study, it is noteworthy that, as there are no instruments aimed at evaluating patients’ knowledge about therapy with warfarin already validated for the Brazilian population, the criterion validity could not be established, as this would have required using a gold standard instrument. Added to this, the fact that although the sample size was sufficient to demonstrate the performance of the instrument, this relatively small number of individuals can also limit the ability to generalize the results for different groups of Brazilian patients on warfarin.

As another limitation of the study, OAK Test was originally designed to be self-administered by individuals with at least seven years of education. However, due to the low level of education presented by several individuals enrolled in this study, we preferred to administer the instrument by patient interview, which has extended the time of application.

However, the use of a rigorous methodological process to evaluate the psychometric properties of the Brazilian version of the OAK Test tried to maintain the reliability and validity of the original instrument in English. In addition, this instrument is useful, quick, and easy-to-apply in clinical practice, by means of individual interviews with patients with different levels of education. Based on a previous publication [12] we identified good content and face validity of the instrument. The instrument was proved to be effective in assessing what it proposes to measure, and that most of the items analyzed are relevant. This finding is in agreement with that of other studies with similar goals [8, 11]. Item 2 of the instrument was a reason of debate among the authors. Most of the population studied uses only the 5 mg warfarin tablet that belongs to the list of drugs distributed by the National Health System in Brazil, and that is not distinguishable by the color that identifies the drug concentration as it happens in the US. Thus, participants may present difficulties in understanding this question. However, we made a decision to retain Item 2 in the final version to provide a broader scope of the instrument to be used not only by public health services but also by private institutions where warfarin brands that allow patients to distinguish different drug concentrations by color may be preferably prescribed.

The results of the test may assist the stratification of patients and the improvement of planning and individualization of educational practices according to the needs of the patients. The need for further studies to check the performance of the instrument in individuals with characteristics different from the study group is indicated.

## Conclusion

The Brazilian version of the OAK Test showed good psychometric properties and was found to be a reliable and valid instrument for evaluating the knowledge of Brazilian patients on oral anticoagulant therapy using warfarin. The results generated with the use of the instrument may be incorporated into the practice of public health care, in order to structure health education activities to ensure improvement in the knowledge of the therapy proposed, which may help increase the effectiveness and safety of the treatment.

The use of the instrument will also aid the analysis of the relationship between the knowledge of the patients and the quality of the anticoagulation treatment using warfarin, as well as the comparison of research findings between different countries. It favors, then, the communication between health professionals and patients, and contributes to the rapid identification of problems and priority needs, as well as to decision-making.

## Abbreviations

ICC, intraclass correlation coefficients; INR, international normalized ratio; OAK, oral anticoagulation knowledge; PT, prothrombin time; R_s_, Spearman correlation; SD, standard deviation; SPSS, Statistical Package for Social Sciences TTR, time within therapeutic range

## References

[1] Ageno W, Gallus AS, Wittkowsky A, Crowther M, Hylek EM, Palareti G (2012). Oral anticoagulant therapy: antithrombotic therapy and prevention of thrombosis, 9th ed: American College of Chest Physicians evidence-based clinical practice guidelines. Chest.

[2] You JJ, Singer DE, Howard PA, Lane DA, Eckman MH, Fang MC (2012). Antithrombotic therapy for atrial fibrillation: Antithrombotic Therapy and Prevention of Thrombosis, 9th ed: American College of Chest Physicians Evidence-Based Clinical Practice Guidelines. Chest.

[3] Whitlock RP, Sun JC, Fremes SE, Rubens FD, Teoh KH (2012). Antithrombotic and thrombolytic therapy for valvular disease: Antithrombotic Therapy and Prevention of Thrombosis, 9th ed: American College of Chest Physicians Evidence-Based Clinical Practice Guidelines. Chest.

[4] Holbrook A, Schulman S, Witt DM, Vandvik PO, Fish J, Kovacs MJ (2012). Evidence-based management of anticoagulant therapy: Antithrombotic Therapy and Prevention of Thrombosis, 9th ed: American College of Chest Physicians Evidence-Based Clinical Practice Guidelines. Chest.

[5] Wiedermann CJ, Stockner I (2008). Warfarin-induced bleeding complications – clinical presentation and therapeutic options. Thromb Res.

[6] Ansell J, Hirsh J, Hylek E, Jacobson A, Crowther M, Palareti G (2008). American College of Chest Physicians Pharmacology and management of the vitamin K antagonists: American College of Chest Physicians Evidence-Based Clinical Practice Guidelines. Chest.

[7] White HD, Gruber M, Feyzi J, Kaatz S, Tse HF, Husted S, Albers GW (2007). Comparison of outcomes among patients randomized to warfarin therapy according to anticoagulant control: results from SPORTIF III and V. Arch Intern Med.

[8] Zeolla MM, Brodeur MR, Dominelli A, Haines ST, Allie ND (2006). Development and validation of an instrument to determine patient knowledge: the oral anticoagulation knowledge test. Ann Pharmacother.

[9] Delgado PG, Garralda MAG, Parejo MIB, Lozano FF, Martínez FM (2009). Validación de un cuestionario para medir el conocimiento de los pacientes sobre sus medicamentos. Aten Primaria.

[10] Baker JW, Pierce KL, Ryals CA (2011). INR goal attainment and oral anticoagulation knowledge of patients enrolled in an anticoagulation clinic in a Veterans Affairs medical center. J Manag Care Pharm.

[11] Matalaqah LM, Radaideh K, Sulaiman SASS, Hassali MA, Kader MASAK (2013). An instrument to measure anticoagulation knowledge among Malaysian community: a translation and validation study of the Oral Anticoagulation Knowledge (OAK) Test. J Pharm Biomed Sci.

[12] Martins MAP, Praxedes MFS, Paiva S, Ribeiro DD, Marcolino MS, Guimarães MHN. Adaptação Transcultural do Oral Anticoagulation Knowledge Test para o português do Brasil. Cienc Saude Colet. 2016:23. [in press]. Available at: http://www.cienciaesaudecoletiva.com.br/artigos/artigo_int.php?id_artigo=15389. Accessed 15 June 2016. 10.1590/1413-81232017225.17782015 28538931

[13] Beaton D, Bombardier C, Guillemin F, Ferraz MB. Recommendations for the Cross- Cultural Adaptation of the DASH & Quick DASH Outcome Measures. Institute for Work & Health. 2007. Available at http://www.dash.iwh.on.ca/sites/dash/files/downloads/cross_cultural_adaptation_2007.pdf. Accessed 1 June 2016.

[14] Roberts P, Priest H, Traynor M (2006). Reability and validity in research. Nurs Stand.

[15] Streiner DL, Norman GR (1989). Health measurement scales: a practical guide to their development and use.

[16] Fayers PM, Machin D (2007). Quality of life: assessment, analysis and interpretation.

[17] Rosendaal FR, Cannegieter SC, van der Meer FJ, Briët E (1993). A method to determine the optimal intensity of oral anticoagulant therapy. Thromb Haemost.

[18] Sijtsma K (2009). On the use, the misuse, and the very limited usefulness of Cronbach’s Alpha. Psychometrika.

[19] Ajzen I, Fishbein M (1998). Understanding Attitudes and Predicting Social Behavior.

[20] Khudair IF, Hanssens YI. Evaluation of patients’ knowledge on warfarin in outpatient anticoagulation clinics in a teaching hospital in Qatar. Saudi Med J. 2010; 31:672–7. 20563367

[21] Mayet AY (2015). Association between oral anticoagulation knowledge, anticoagulation control, and demographic characteristics of patients attending an anticoagulation clinic in Saudi Arabia: a cross-sectional prospective evaluation. Trop J Pharm Res.

[22] Alphonsa A, Sharma KK, Sharma G, Bhatia R (2015). Knowledge regarding oral anticoagulation therapy among patients with stroke and those at high risk of thromboembolic events. J Stroke Cerebrovasc Dis.

[23] Janoly-Duménil A, Bourne C, Loiseau K, Luauté J, Sancho PO, Ciancia S (2011). Oral anticoagulant treatment - evaluating the knowledge of patients admitted in physical medicine and rehabilitation units. Ann Phys Rehabil Med.

[24] Gras-Champel V, Voyer A, Lematte C, Pakula P, Roussel B, Lefrère JJ, Andréjak M (2005). Assessment of the quality of oral anticoagulation management in patients admitted to Amiens University Hospital. Therapie.

[25] Hasan SS, Shamala R, Syed IA, Basariah N, Chong DW, Mei TK, Chin OH (2011). Factors affecting warfarin-related knowledge and INR control of patients attending physician- and pharmacist-managed anticoagulation clinics. J Pharm Pract.

